# Pennogenyl Saponins from *Paris quadrifolia* L. Induce Extrinsic and Intrinsic Pathway of Apoptosis in Human Cervical Cancer HeLa Cells

**DOI:** 10.1371/journal.pone.0135993

**Published:** 2015-08-21

**Authors:** Justyna Stefanowicz-Hajduk, Rafal Bartoszewski, Sylwia Bartoszewska, Kinga Kochan, Anna Adamska, Igor Kosiński, J. Renata Ochocka

**Affiliations:** 1 Department of Biology and Pharmaceutical Botany, Medical University of Gdansk, Gdansk, Poland; 2 Department of Inorganic Chemistry, Medical University of Gdansk, Gdansk, Poland; Karolinska Institutet, SWEDEN

## Abstract

Pennogenyl saponins are the active compounds of large number of plant species and consequently many polyherbal formulations. Hence, great interest has been shown in their characterization and in the investigation of their pharmacological and biological properties, especially anticancer. This present study reports on the evaluation of cytotoxic effects and explanation of the molecular mechanisms of action of the two pennogenyl saponins (PS 1 and PS 2) isolated from *Paris quadrifolia* L. rhizomes on human cervical adenocarcinoma cell line HeLa. To determine the viability of the cells treated with the compounds we used real-time cell proliferation analysis and found that the pennogenyl saponins PS 1 and PS 2 strongly inhibited the tumor cells growth with IC50 values of 1.11 ± 0.04 μg/ml and 0.87 ± 0.05 μg/ml, respectively. The flow cytometry analysis indicated that the two compounds induced apoptosis in a dose-dependent manner and decreased mitochondrial membrane potential in HeLa cells in the early stage of apoptosis. Quantitative PCR and Western Blot analysis showed that the two saponins significantly increased mRNA expression of FADD and BID as well as induced caspase-8 via increased of procaspase-8 processing in the treated cells. The results of this study suggest that both the extrinsic death receptor and intrinsic mitochondrial pathways are involved in the programmed cell death.

## Introduction

Steroidal saponins are the group of secondary metabolites which are found in great number of monocotyledonous plants. Consequently, they are constituents of many plant drugs and folk medicines, especially of Orient origin [1] where common sources of saponins are the species from the *Liliaceae* family. One of the important saponin-bearing genus from this family is *Paris*, that includes over twenty plant species. These species contain mainly pennogenyl and diosgenyl glycosides [2] which are physiologically active compounds [3, 4] and play an important role in the treatment of neoplasms, hemostatic disturbances, inflammation and fungal infection [5–7]. In folk medicine, they are beneficial in the treatment of traumatic injuries, snake bite, abscess, parotitis and mastitis.

Much attention is paid to the cytotoxic activity of pennogenin saponins isolated from *Paris polyphylla* [8–10]. These components show significant antiproliferative activities on liver, breast and prostate cancer cells [11, 12]. Recent data indicate that pennogenyl glycosides possess an anti-metastatic effect on melanoma cells [13] and *in vivo* anticancer activity towards hepatocellular carcinoma [14]. The strength of these effects on tumor cells is diverse and is strictly connected with chemical structure of saponin compounds which is mostly well known [10, 15–17].

Despite the numerous phytochemical studies, there is quite few research which attempt to explore the mechanisms of pennogenyl saponins action on tumor cells, mainly due to their low contents in plants [9, 13, 14].

The present study investigates the mechanism of cytotoxic effects of the two pennogenyl saponins (PS) isolated from *Paris quadrifolia* L. on human cervical adenocarcinoma cells (HeLa). The saponins were obtained from the *Paris* rhizomes and chemically identified in our previous study [18]. The structure of compound **PS 1** was determined as pennogenin 3-*O*-α-L-rhamnopyranosyl-(1→4)-[α-L-rhamnopyranosyl-(1→2)]-β-D-glucopyranoside

and compound **PS 2** as pennogenin 3-*O*-α-L-rhamnopyranosyl-(1→4)-α-L-rhamnopyranosyl-(1→4)[α-L-rhamnopyranosyl-(1→2)]-β-D-glucopyranoside. These compounds have strong antitumor properties on the tested cells. However, the mechanisms of their cytotoxic action, according to our knowledge, remain partially elucidated [19]. One of the possible mechanism that plays a role in inhibition of HeLa cells proliferation is induction of apoptosis via the activation of caspases related to the intrinsic signaling pathway [19]. In our study, we postulate that both the pennogenyl saponins induce apoptosis also through the activation of the death receptor-mediated pathway.

## Materials and Methods

### Materials

Dulbecco’s Modified Eagle’s Medium (DMEM), penicillin, streptomycin, L-glutamine, DMSO, PBS, 3-(4,5-dimethylthiazol-2-yl)-2,5-diphenyltetrazolium bromide (MTT) dye were purchased from Sigma-Aldrich (St. Louis, MO, USA).

The isolation and chemical characteristics of the two examined pennogenyl saponins from *P*. *quadrifolia* rhizomes were performed and described previously [18]. The lyophilized compounds were dissolved in DMSO at a concentration of 1 mg/ml.

### Cell line culture

The human cervical adenocarcinoma cell line (HeLa S3) and human keratinocytes (HaCaT) were obtained from the American Type Culture Collection (ATCC, USA). Cell lines were cultured in DMEM supplemented with 10% (v/v) FBS, 100 units/ml of penicillin, 100 μg/ml of streptomycin, 2 mM L-glutamine, and were kept at 37°C in a humidified 5% CO_2_ incubator.

### MTT assay

The viability of the cells was determined using the MTT assay. The cells were seeded in 96-well plates at a density of 2x10^3^ cells/well and treated for 24 h with the compounds PS 1 and PS 2 in the concentration range of 0.1–10.0 μg/ml. DMSO was added to the control cells at a final concentration of 1.0% (v/v), which was related to the maximal concentration of the solvent compounds used in the experiment. Following treatment, MTT (0.5 mg/ml) was added to the medium and cells were incubated for 3 h at 37°C. The absorbance of the formazan solution was measured at 570 nm with a plate reader (Epoch, BioTek Instruments, USA). The results are expressed as IC50 mean values (±SD, standard deviation) of at least two independent experiments.

### xCELLigence cell proliferation assay

For real-time monitoring of cell viability, we used the xCELLigence system (ACEA Biosciences, USA). The cells were seeded at a density of 2x10^4^/well into E-plate 16 (ACEA Biosciences, USA) containing 100 μl medium per well. When the cells entered log phase, the compounds PS 1 and PS 2 were added at final concentrations of 0.1–10.0 μg/ml. A final DMSO concentration in the wells did not exceed 1.0% (v/v). The cells were incubated with the compounds and monitored for 24 h at 37°C in a 5% CO_2_ atmosphere. The RTCA software v. 1.2.1 was used to calculate the half maximal inhibitory concentration (IC50) values. All experiments were performed in duplicate, in three independent repeats.

### Trypan blue assay

The cells (1x10^5^ cells/well) were incubated with the tested compounds at a concentration of 1.0–5.0 μg/ml. After 24 h the cell viability was determined using 0.2% (v/v) trypan blue solution (final concentration) and cell counter (Countess Automated Cell Counter, Life Technologies, USA). The experiments were repeated at least two times.

### Hoechst staining for apoptosis analysis

The apoptotic effect of the compounds was analyzed by using the blue fluorescent Hoechst 33342 dye (Life Technologies). HeLa cells were seeded in 6-well plates at a density of 5x10^5^/well. The cells were treated with the compounds PS 1 and PS 2 dissolved in DMSO at a final concentration of 1 μg/ml. DMSO concentration did not exceed 0.1% (v/v). After 24 h the cells were stained with final concentration of 0.5 μg/ml of the dye in PBS for 25 minutes at a CO_2_ incubator and then observed under a fluorescent microscope (Leica, Switzerland).

### DNA extraction and fragmentation assay

HeLa cells were seeded at a density of 5x10^6^ cells into 100 mm culture dishes and treated with the compounds at a final concentration of 1.0 μg/ml. After 48 h the cells were harvested and lysed in lysis buffer (1% NP-40 in 20 mM EDTA, 50 mM Tris-HCl, pH 7.5). After the cell lysis, supernatant was collected and treated with 1% SDS and RNase A (Sigma-Aldrich) at a final concentration of 50 μg/ml. The cells were incubated for 2 h at 56°C. Next, proteinase K (2.5 μg/ml) was added to the supernatant and samples were incubated for 2 h at 37°C. DNA was precipitated with 3M ammonium acetate and ice-cold ethanol at a final concentration of 70% (v/v). The obtained DNA pellet was dissolved in TE buffer (10 mM Tris, 1 mM EDTA, pH 8.0) and subjected to electrophoresis on 1.5% agarose gel with ethidium bromide.

### Apoptosis assay

Induction of apoptosis was assessed by the binding of annexin V-phycoerythrin/7-amino-actinomycin (PE/7-AAD) to cellular phosphatidylserine. The cells (1x10^5^) were treated with the two compounds at a concentration of 0.5–5.0 μg/ml for 24 h. These concentrations values of the saponins were chosen for showing significant changes in the number of the apoptotic cells. The concentration of DMSO as a control sample did not exceed 0.5% (v/v). The cells were harvested and stained using Muse Annexin V and Dead Cell Assay Kit (Merck Millipore, Germany), following the protocol provided by the manufacturer. Apoptotic cells were then analyzed by Muse Cell Analyzer (Merck Millipore). The experiments were repeated at least two times.

### Assessment of mitochondrial membrane potential

Hela cells were seeded at a density of 1x10^5^ cells/well and treated with the two compounds at a concentration of 1.0–10.0 μg/ml. These concentrations values were chosen for indicating the mitochondrial dysfunction in the early stage of the cell treating. The concentration of DMSO as a control sample did not exceed 1% (v/v). After 3 h of exposure, the cells were harvested and prepared using Muse MitoPotential Kit (Merck Millipore) according with the manufacturer’s protocol. The percent of depolarized/live cells was determined by Muse Cell Analyzer. All the experiments were repeated at least two times.

### Real-time PCR

The synthesis of cDNA and real-time PCR reactions were performed as described previously [20]. Briefly, total RNA was isolated by using RNeasy Mini Kit (Qiagen, The Netherlands) following the manufacturer’s instructions. The concentration of RNA was measured and cDNA synthesis was performed using Maxima First Strand cDNA Synthesis Kit (Thermo Scientific, USA), according with the manufacturer’s protocol.

#### Real-time PCR gene expression arrays

cDNA obtained from the cells treated with the compounds as well as from the cells treated with control vehicle, were applied on The Applied Biosystems TaqMan Array Human Apoptosis 96-well Plates (Life Technologies 4414072). Each plate contains 88 assays for apoptosis associated genes and 4 assays for candidate endogenous control genes. The PCR reactions were set according to the manufacturer’s instructions and performed on ABI7500 Real Time PCR system. The resulting data were analyzed with ABI 2.05 software based on the comparative dCT method [21]. To validate arrays results, we independently analyzed (with RT-PCR) all significantly changed transcripts as well as genes with unaffected levels.

#### Measurement of expression using quantitative real-time PCR (qRT-PCR)

To further confirm expression of specified genes, we used specific TaqMan probes (BAX assay id Hs00180269_m1; BCL2 assay id Hs00608023_m1; GAPDH assay id Hs02758991_g1; 18S rRNA assay id Hs99999901_s1; FADD assay id Hs04187499_m1; BID assay id Hs00609632_m1; TNFSF10 assay id Hs00921974_m1; DEDD2 assay id Hs00370206_m1; BAD assay id Hs00188930_m1) and TaqMan One-Step RT-PCR Master MixReagents (Life Technologies) as described previously [22]. The resulting data were analyzed with ABI 2.05 software based on the comparative relative standard curve method [23].

### Western Blot Analysis

Hela cells were treated with the two compounds PS 1 and PS 2. After treatment, the cells were harvested and lysed in RIPA buffer (150 mM NaCl, 1% NP-40, 0.5% sodium deoxycholate, 0.1% SDS, 50 mM Tris-HCl, pH 8.0). The total protein concentration was determined by Bio-Rad Protein Assay (Bio-Rad Laboratories, USA). The samples were separated electrophoretically using prestained SDS PAGE gels (Bio-Rad Laboratories) and transferred to a polyvinylidene fluoride (PVDF) membranes. The membranes were kept at 4°C overnight in 3% BCA (Sigma-Aldrich) in PBS/Tween-20 and then incubated with the primary antibodies at one of the following: β-actin (ab 1801, Abcam, UK), Bid (ab 32060, Abcam), Bcl-2 (ab 59348, Abcam), caspase-8 (sc-81661, Santa Cruz Biotechnology, USA). After washing with PBS/Tween-20, the membranes were incubated with goat anti-rabbit IgG or with goat anti-mouse IgG HRP conjugated secondary antibodies (Bio-Rad Laboratories). Immunodetection was performed with an enhanced chemiluminescence (ECL) detection kit (Bio-Rad Laboratories). The protein brands were analyzed using ChemiDoc system equipped with Image Lab software v. 4.1 (Bio-Rad Laboratories).

### Statistical analysis

All data are expressed as mean values ± standard deviation (SD). Statistical comparisons of the results were evaluated using the Student’s t-test.

## Results

### The saponin compounds PS 1 and PS 2 decreased HeLa and HaCaT cells viability

The effect of the *P*. *quadrifolia* pennogenyl saponins on the cells viability was examined by the xCELLigence system which is based on electronic impedance measurement of sensor electrodes in E-plate wells and allows continuous, quantitative monitoring of cells [24]. Changes in cells viability, number, morphology and degree of adhesion affect electrode impedance which is described by a parameter called Cell Index (CI) [25, 26]. The CI is used for calculating the IC50 value in every measuring point of the experiment.

HeLa cells were treated with the compounds PS 1 and PS 2 (0.1–10.0 μg/ml) for 24 h. The system allowed to assess IC50 values for each of the compounds. The exposure of HeLa cells to the saponins resulted in a significant decrease in the cell viability. After treatment with the compound PS 1 and PS 2, the obtained IC50 values were 1.11 ± 0.04 μg/ml and 0.87 ± 0.05 μg/ml, respectively (Table 1).

**Table 1:** IC50 values (μg/ml) of the pennogenyl saponins (PS 1 and PS 2) based on the xCELLigence system (RTCA) and MTT assay.

Pennogenyl saponins	HeLa cells		HaCaT cells	
	RTCAIC50 [μg/ml]	MTT assayIC50 [μg/ml]	RTCAIC50 [μg/ml]	MTT assayIC50 [μg/ml]
PS 1	1.11 ± 0.04, R^2^ ^a^ = 0.94	0.93 ± 0.15	1.01 ± 0.01, R^2^ = 0.98	0.82 ± 0.13
PS 2	0.87 ± 0.05, R^2^ = 0.98	0.55 ± 0.01	0.94 ± 0.04, R^2^ = 0.97	0.58 ± 0.04

^a^R^2^- the coefficient of determination

The IC50 values from the xCELLigence system were obtained based on the sigmoidal dose-response formula and calculated from repeated experiments (n = 3). To confirm the results from the xCELLigence system, we performed MTT assay using the same concentration range of the two saponins (0.1–10.0 μg/ml) for HeLa and HaCaT cells. The cells were treated for 24 h and the recorded IC50 values for the compounds were obtained from three independent experiments (Table 1).

**Fig 1:**
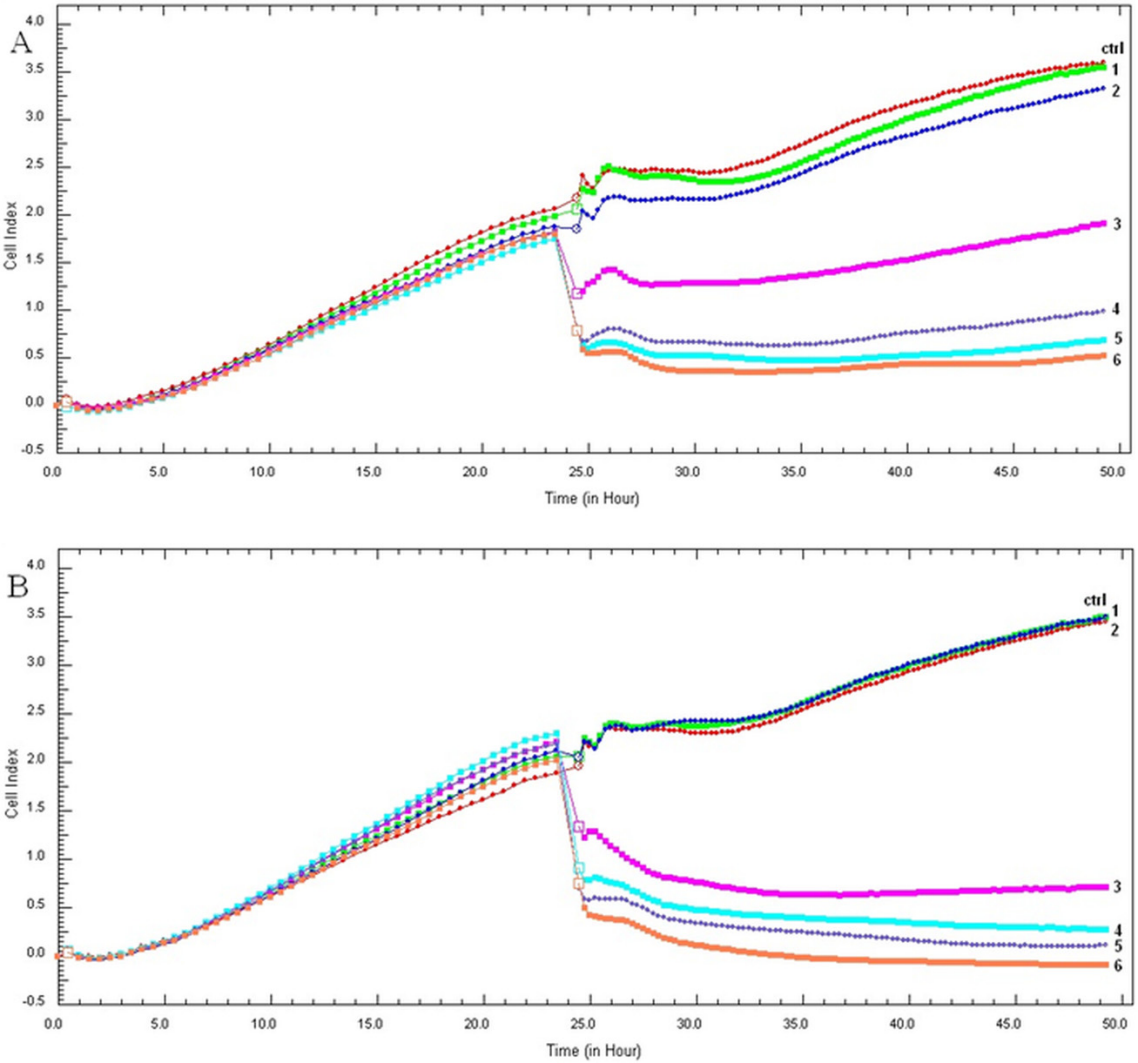
The pennogenyl saponins treatment decreased HeLa cells viability. The cells were incubated with the pennogenyl saponins PS 1 (A) and PS 2 (B) at different concentrations for 24 h. The numeric labels on the curves represent the increasing concentration values of the compounds (0.1, 0.5, 1.0, 2.0, 5.0, 10.0 μg/ml, respectively).

**Fig 2:**
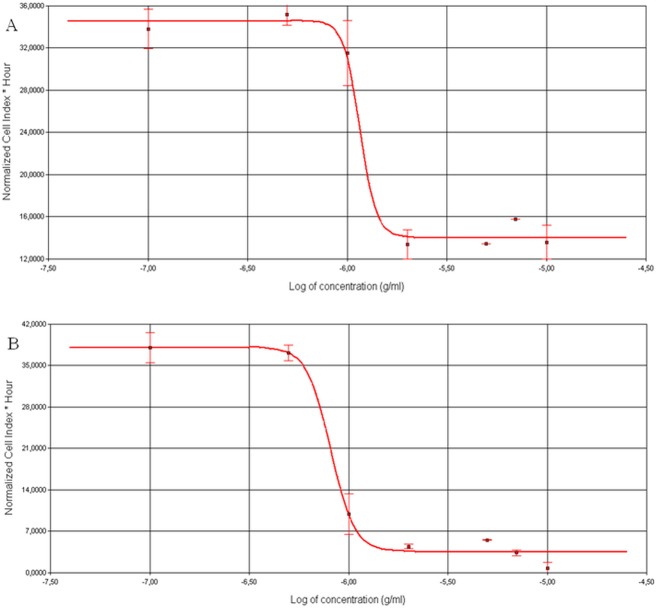
RTCA measurement of CI values of HeLa cells treated with the compound PS 1 (A) and PS 2 (B) for 24 h. The IC50 values of the saponins were calculated based on the dose-response curves of the cell index by the xCELLigence system. Error bars represent the standard deviations.

The used control sample with DMSO had no inhibition effect on the cells. Our results show that the two pennogenyl saponins had a dose-dependent and time-dependent activity (Fig 1 and Fig 2). The viability of HeLa cells decreased during the incubation of the cells with the compounds. Significantly changes in the cells adhesion were observed after 5 h of treatment the cells with the saponins. The cells viability also reduced together with higher concentrations of PS 1 and PS 2.

The same experiments were performed on non-tumor control cell line–human keratinocytes. The IC50 values were 1.01 ± 0.01 μg/ml for the compound PS 1 and 0.94 ± 0.04 μg/ml for PS 2 (Table 1).

Real-time cellular analysis and MTT assay indicated stronger cytotoxic effect of the saponin PS 2 on the studied cells. Furthermore, this effect was confirmed by trypan blue exclusion assay.

### Effect of the saponins-induced cell apoptosis

**Fig 3:**
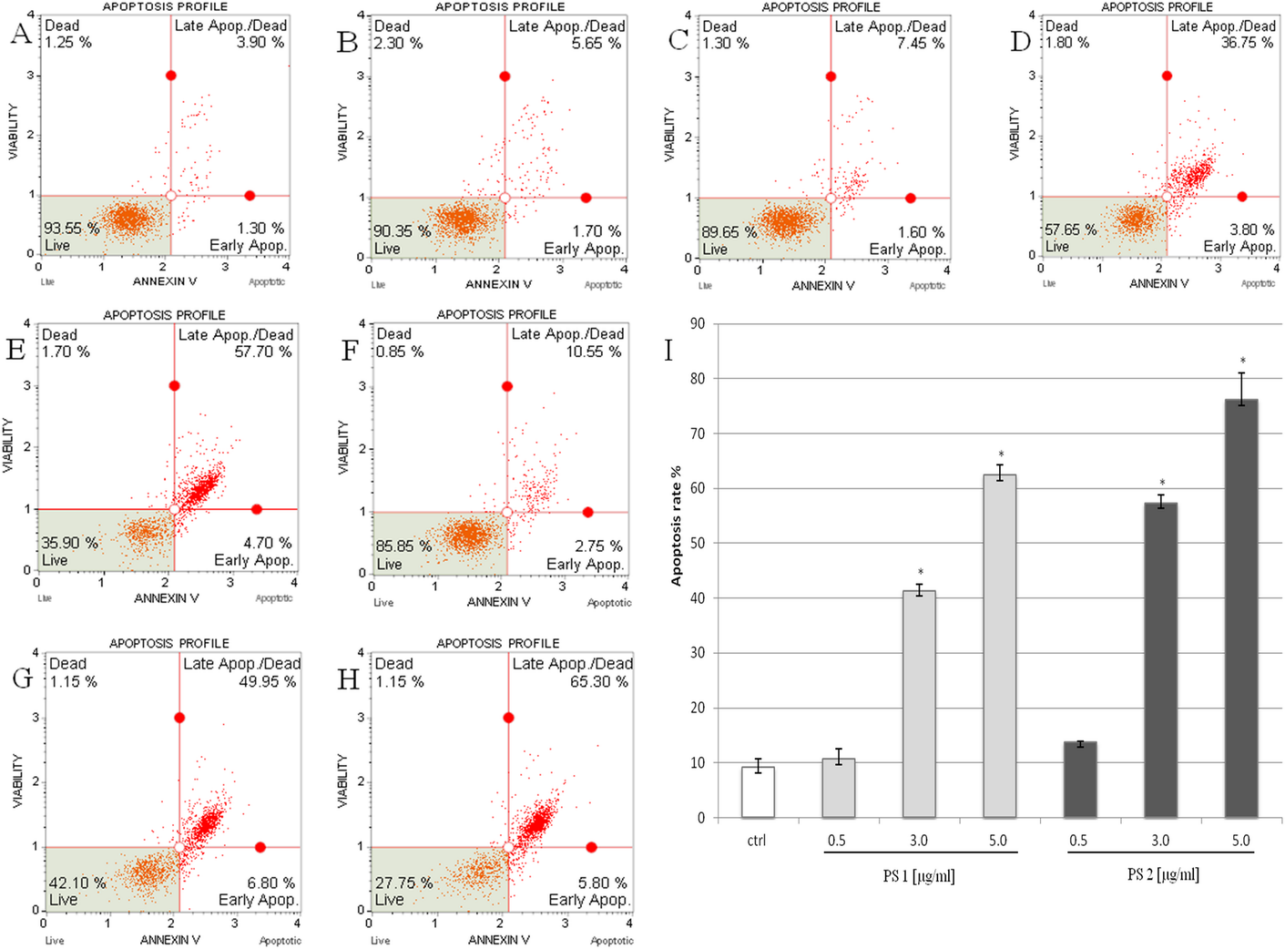
The pennogenyl saponins induced apoptosis in HeLa cells. The cells were analyzed by flow cytometry using annexin V-PE/7-AAD staining method. The distribution of the apoptotic cells was determined in the sample of untreated cells (A) and after incubating the cells with 0.5% DMSO (B), the compound PS 1 (C-E) and PS 2 (F-H) at a concentration of 0.5, 3.0 and 5.0 μg/ml, respectively. The cells were treated for 24 h and the total extent of apoptosis (apoptosis rate) was determined in comparison to the DMSO control (I). Each sample was run in triplicate. Error bars represent standard deviations. Significant differences relative to the control are marked with an “*” (p<0.05).

To confirm the apoptotic effect of the two pennogenyl compounds, HeLa cells (after being treated for 24 h) were stained with annexin V-PE/7-AAD and analyzed by Muse analyzer. The percentage of apoptotic cells increased in a dose-dependent manner. The total apoptotic rates (the total percentage of early and late apoptotic cells) for the compound PS 1 were 10.71%, 41.35% and 62.48% for concentrations 0.5, 3.0 and 5.0 μg/ml, respectively. The apoptotic rates for the compound PS 2 were 13.80%, 57.37%, 76.12% for concentrations 0.5, 3.0 and 5.0 μg/ml, respectively (Fig 3).

**Fig 4:**
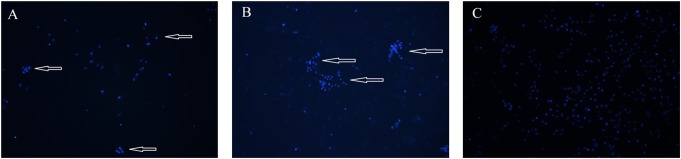
The pennogenyl saponins induced apoptotic changes in HeLa cells. Nuclear chromatin state was assessed with Hoechst 33342 staining after exposure the cells to 1 μg/ml of the compound PS 1 (A) and PS 2 (B) for 24 h (the concentration value related to the IC50 values of the saponins). The cells treated with the saponins show condensed chromatin relative to the control cells incubated with 0.1% DMSO (C). Arrows represent apoptotic cells.

To estimate the changes in chromatin distribution within the cells treated with the two compounds, we did the visualization of the cellular nuclei with Hoechst 33342 dye. After staining, the chromatin condensation and nuclear fragmentation in HeLa cells were observed as compared with the control cells incubated with DMSO (Fig 4). The experiment confirmed induction of apoptosis in the cells treated with the saponins.

**Fig 5:**
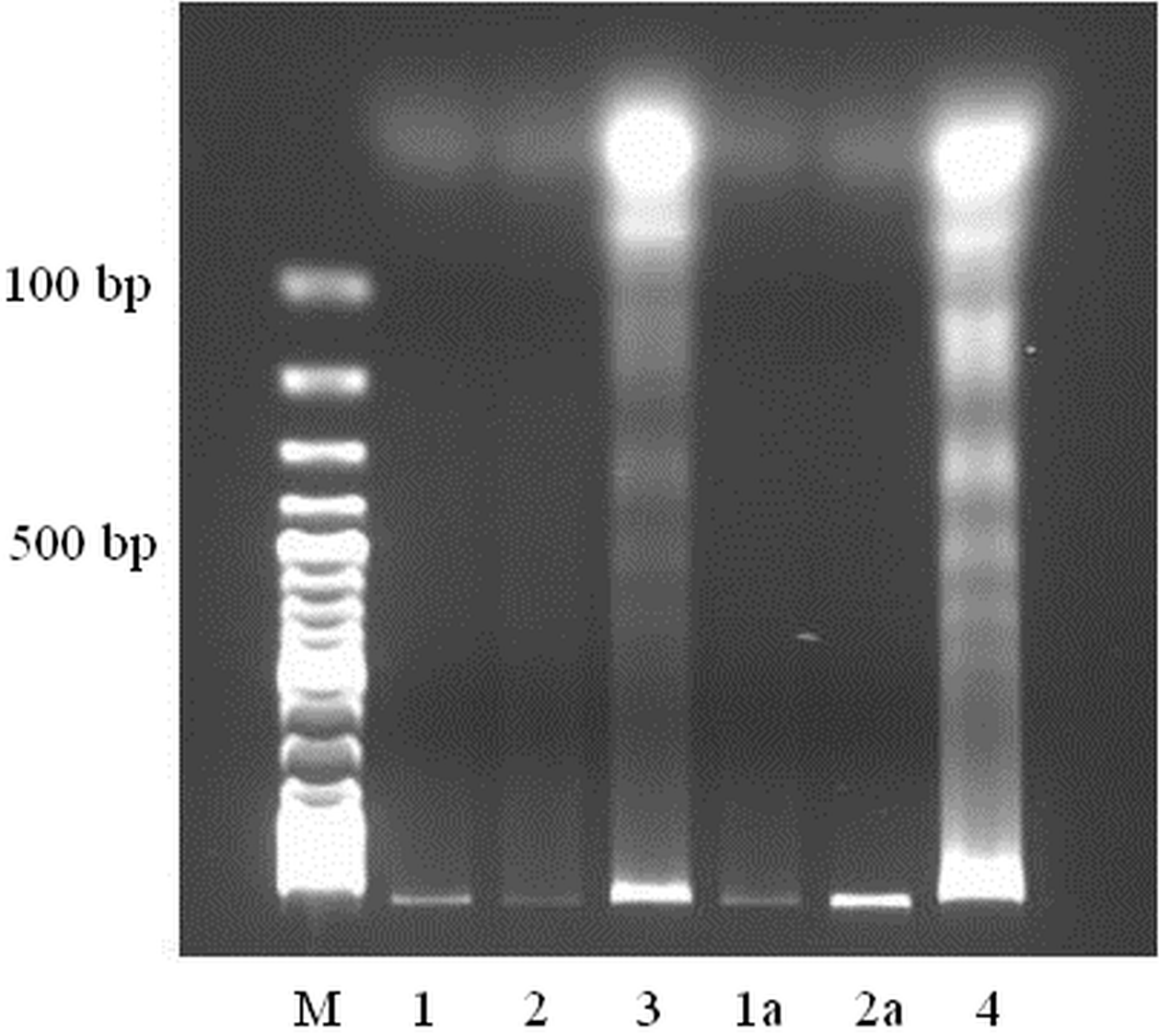
The saponins treatment induced DNA fragmentation in HeLa cells. Lanes 1 and 1 a represent DNA of untreated cells; lanes 2, 2a – cells treated with 0.1% DMSO (ctrl); lane 3 – cells treated with the compound PS 1; lane 4—cells treated with the compound PS 2. The cells were incubated with the compounds at a concentration of 1 μg/ml for 48 h (the concentration value related to the IC50 values of the compounds). M–DNA ladder (Thermo Scientific).

Furthermore, the results present in Fig 5 show the undamaged DNA in the control cells whereas the compounds treated cells exhibited DNA fragmentation characteristic for apoptotic nuclei.

### The pennogenyl compounds PS 1 and PS 2 modulated mitochondrial membrane potential (ΔΨm) of HeLa cells

**Fig 6:**
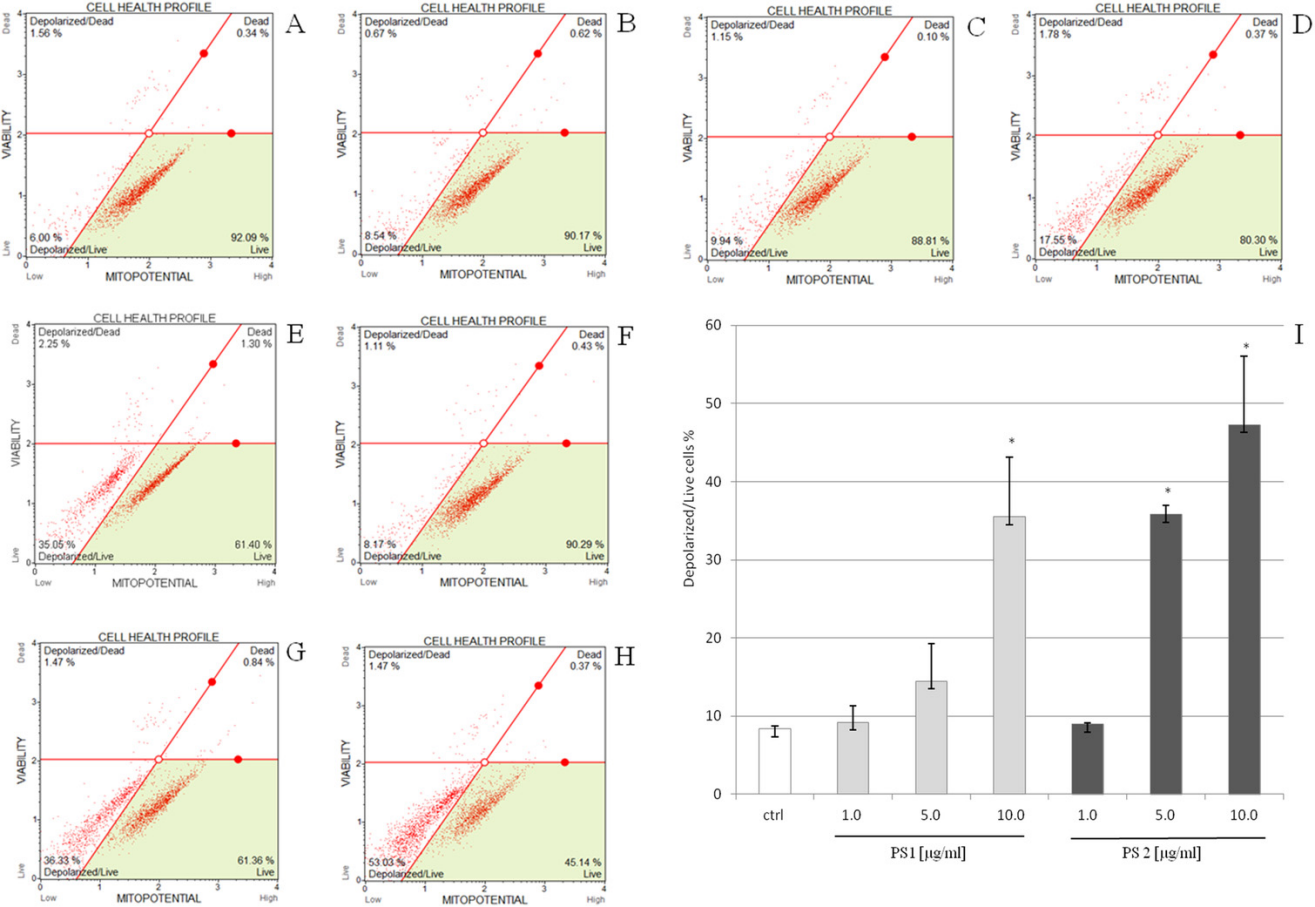
Pennogenyl saponins-induced changes in transmembrane mitochondrial potential in HeLa cells as determined by flow cytometry. The distribution of the cells undergoing loss of mitochondrial potential was determined after incubating the untreated cells (A), the cells with 1.0% DMSO (B), the compound PS 1 (C-E) and PS 2 (F-H) at a concentration of 1.0, 5.0, 10.0 μg/ml, respectively. The cells were treated for 3 h and the extent of mitochondrial cell depolarization was determined in comparison to the DMSO control (I). Each sample was run in triplicate. Error bars represent standard deviations. Significant differences relative to the control are marked with an “*” (p<0.05).

Loss of the mitochondrial inner transmembrane potential is a reliable indicator of mitochondrial dysfunction and cellular health. This effect is often observed to be associated with the early stages of apoptosis. After treating HeLa cells with the studied compounds, the state of mitochondrial membranes of the cells was estimated by Muse system. The control cells showed high fluorescence due to accumulation of the fluorescent dye within inner membrane of intact mitochondria. The cells incubated with increasing concentrations of the saponins demonstrated a decrease in fluorescence. The percentage of depolarized/live cells treated with the compound PS 1 was 9.20%, 14.48%, 35.52% for concentrations 1.0, 5.0 and 10.0 μg/ml, respectively. The values for the compound PS 2 were 8.98%, 35.82%, 47.26% for concentrations 1.0, 5.0 and 10.0 μg/ml, respectively (Fig 6). The obtained results show that mitochondria play a role in induction of apoptosis in the cells treated with the two examined saponins.

### The two pennogenyl saponins increased mRNA expression of FADD and BID in HeLa cells

**Fig 7:**
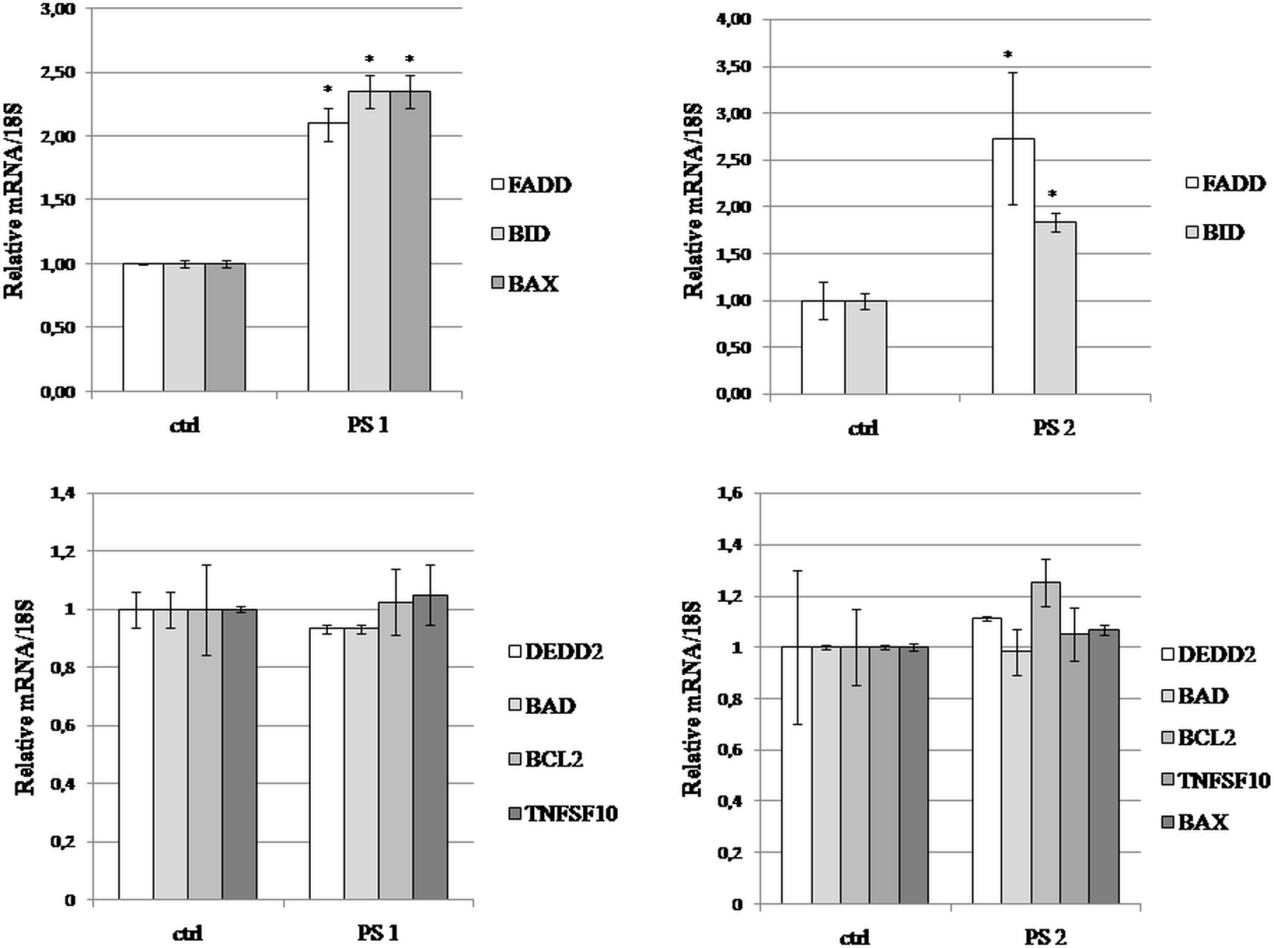
The pennogenyl compounds treatment induced changes in mRNA levels of the apoptotic genes in HeLa cells. The cells were stimulated with the compounds PS 1 and PS 2 at a concentration of 1.0 and 0.5 μg/ml, respectively and incubated for 24 h. The mRNA levels were measured in real-time PCR experiments. Each sample was run in duplicate, and the relative amount of mRNA was normalized to the 18S rRNA content and expressed as a fold-change over the control (0.1% DMSO). Error bars represent standard deviations. Significant differences relative to the control are marked with an “*” (p<0.05).

To determine the extent of the tested compounds on apoptotic pathways, the expression of 88 related genes was tested with qPCR (qPCR human apoptosis array). Among the examined assays from this array, for further experiments two groups of genes were chosen–the genes whose expression was significantly changed and the ones with expression comparable to the control. To confirm the results of the PCR arrays, the real-time quantitative PCR was used. The analysis show changes of mRNA expression of Fas-associated death domain (FADD) and BH3 interacting domain death agonist (BID) after treatment with the two saponins (Fig 7). Additionally, the mRNA level of BCL2 associated X protein (BAX) significantly increased in HeLa cells incubated with the compound PS 1 for 24 h. Furthermore, as shown in Fig 7, the mRNA expression of death effector domain containing 2 (DEDD2), B-cell lymphoma protein 2 (BCL2), BCL2 antagonist of cell death (BAD) and tumor necrosis factor (ligand) superfamily, member 10 (TNFSF10) was comparable with the control.

### Effects of the saponins on the protein expression of Bcl-2, Bid and procaspase-8

**Fig 8:**
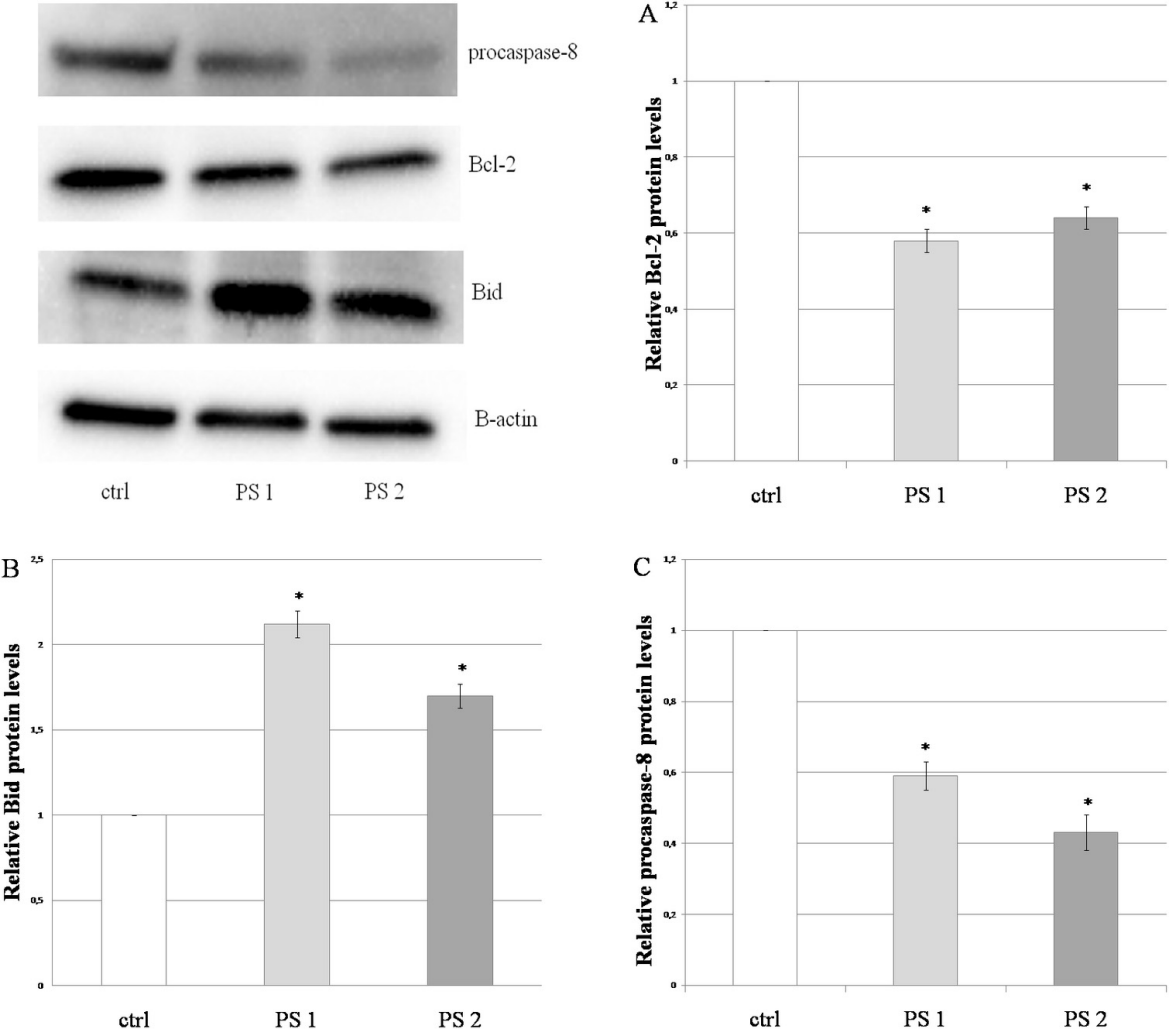
The pennogenyl saponins treatment induced changes in apoptosis-related protein expression. HeLa cells were incubated with the compound PS 1 and PS 2 at a concentration of 1.0 μg/ml (the concentration value related to the IC50 values of the saponins) for 5 h and the expression of proteins in the treated cells was determined by Western blot analysis. PS 1 and PS 2 decreased Bcl-2 (A) and procaspase-8 (55 kDa) (C) protein levels and increased Bid (B) protein expression in the cells. The experiments were repeated three times and each protein level was related to 0.1% DMSO control sample. Error bars represent standard deviations. Significant differences relative to the control are marked with an “*” (p<0.05).

To further elucidate the mechanism of apoptosis in HeLa cells treated with the compounds, we examined the protein expression of Bcl-2, Bid and procaspase-8. The cells were incubated with the saponins at a concentration of 1 μg/ml and the control sample (0.1% DMSO) for 5 h (point of time when the changes in the protein levels were significant). The expression of proteins was detected by Western blot. *β*-actin was used as an internal loading control. The obtained results show that the saponins treatment decreased the protein level of Bcl-2 (Fig 8A), whereas Bid expression was increased in comparison to the control sample (Fig 8B). To confirm the role of the extrinsic pathway of apoptosis induced by the compounds, the changes of procaspase-8 expression level were detected. Fig 8C shows the significant decrease in the protein level in the treated HeLa cells.

## Discussion

*Paris* rhizomes have long been used by Chinese people as a remedy for bleeding, microbial infection and inflammation [27, 28]. In addition, the rhizomes have been widely applied in cancer prevention and therapy in traditional medicine [29]. The numerous studies have shown that the extracts from *Paris* species and their main active components–steroidal saponins possess antitumor properties against different cell lines [11, 19, 29–34]. However, only few papers, which attempt to explore the mechanisms of cytotoxic effect of pennogenyl saponins, have been published so far [11, 14, 19].

**Fig 9:**
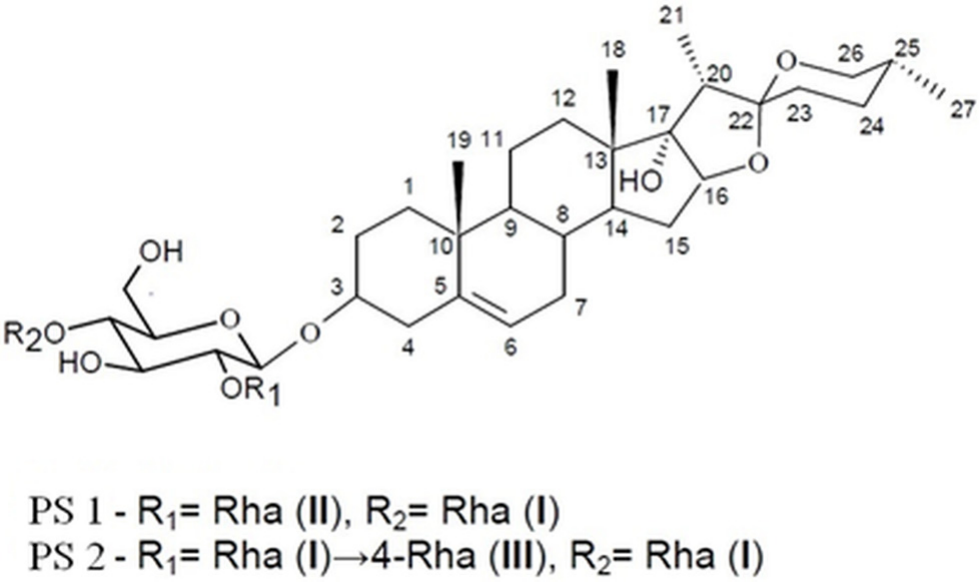
The structure of *Paris* saponin PS 1 and PS 2.

In this study, we examined the effect of the two *P*. *quadrifolia* pennogenyl saponins on human cervical cancer cell line HeLa. The tested compounds exhibited significant antiproliferative activity against the human cells. The results obtained in the experiment with RTCA system show that both saponins demonstrate a dose-dependent and time-dependent inhibitive effect on proliferation of HeLa cells. The compound PS 2 has slightly stronger activity than PS 1, which may be caused by differences in the molecule structures of the saponins (one additional rhamnose residue in the PS 2 sugar chain. Fig 9). The analyses of the relationship between the bioactivity of saponins and chemical structure indicate that a sugar part plays a role in the activity of steroid molecule [32, 35, 36]. Saponins with the same steroidal moiety exhibit different anticancer effect. Compounds with more sugar moieties have stronger activity against cells [12].

The experiments carried out to determine kind of cell death indicate that the two pennogenyl saponins induced apoptosis. Apoptosis is characterized by typical morphological and biochemical hallmarks, including cell shrinkage, nuclear DNA fragmentation and membrane blebbing [37]. All these changes were observed in the cells incubated with the two tested pennogenyl saponins. The analysis of the cells treated with the compounds and stained with the fluorescent dye shows nuclear fragmentation and formation of condensed nuclei. Our data obtained in the experiment with electrophoretic separation of extracted DNA from the treated cells support these observations.

Early hallmark of apoptosis is the loss of plasma membrane asymmetry where molecules of phosphatidylserine are translocated from the inner to the outer surface of the cell membrane so that a dependent phospholipid-binding protein (annexin V) could readily bind them [38–40]. In our study, we observed significant increase in population number of apoptotic HeLa cells incubated with the examined saponins and this effect exhibited a dose-dependent pattern.

The main pathways inducing apoptosis are the extrinsic death receptor pathway and intrinsic mitochondria pathway [41–43]. The extrinsic signaling pathways are related to the membrane death receptors that belong to the tumor necrosis factor (TNF) receptor gene superfamily [44, 45]. To date, the fatty acid synthetase ligand/receptor (FasL/FasR) and TNF-α/TNFR1 models are best characterized ones. The linking of FasL to FasR results in induction of apoptosis in sensitive cells through recruitment of adaptor molecular FADD and a FADD-associated procaspase-8 to the death receptor [45–49]. A death inducing signaling complex DISC forms, leading to cell death [50]. Our data show that the two pennogenyl compounds significantly increased mRNA expression of FADD in the treated cells. Additionally, the compound PS 1 and PS 2 induced caspase-8 as shown via increased of procaspase-8 processing in the compounds treated HeLa cells. The activation of caspase-8 generated at the DISC is sometimes insufficient to generate a caspase signaling cascade strong enough for execution of cell death. In this case, the signal requires amplification via mitochondria-dependent apoptotic pathways [51]. The protein that links the caspase signaling cascade and the mitochondria is the Bcl-2 family member Bid. The activated form of Bid (tBid) translocates to the mitochondria where it acts with the proapoptotic proteins Bax and Bak to initiate the release of proapoptotic factors from the mitochondria [52]. In our study, we observed high increase in BID mRNA and protein expression in HeLa cells incubated with the tested compounds. Interestingly, mRNA expression of BAX was much higher for the PS 1-treated cells.

The action of Bid and Bax is counteracted by the antiapoptotic Bcl-2 family member such as Bcl-2 which can inhibit mitochondrial proapoptotic events [53]. The Bcl-2 family of proteins influences on mitochondrial membrane permeability [41, 45, 53] and participates in the formation of pores in the membrane. Permeability transition (PT) is always followed by the disruption of the mitochondrial inner transmembrane potential ΔΨm [54, 55]. Loss of the potential is often observed to be associated with the early stages of apoptosis [56–58]. Our analysis shows that the Bcl-2 protein expression decreased in the cells treated for both the compounds.

Furthermore, our experiments show that the two pennogenyl saponins depolarized the membrane potential significantly in HeLa cells in a dose-dependent manner. This confirms the involvement of the intrinsic mitochondria pathway in the cell apoptosis.

In this study, we analyzed the mechanisms of antitumor action of the two saponins and conclude that both the extrinsic death receptor pathway and intrinsic pathway play a role in the apoptotic effect of the compounds in HeLa cells. The particular signal pathways of the cell death require further investigation especially as the two pennogenyl components isolated from *P*. *quadrifolia* rhizomes could be a potential drug in the treatment of human cervical cancer.
